# Supplementary material for: A fault-intrusion-tolerant system and deadline-aware algorithm for scheduling scientific workflow in the cloud
Source: PeerJ Comput Sci. 2021 Nov 2;7:e747. doi: 10.7717/peerj-cs.747 (PMC8576554; doi:10.7717/peerj-cs.747)
Supplement: Supplemental Information 6 [file peerj-cs-07-747-s006.zip › Myproject1.1/docs/org/workflowsim/clustering/balancing/BalancedClustering.html]

BalancedClustering


JavaScript is disabled on your browser.


- Overview
- Package
- Class
- Use
- Tree
- Deprecated
- Index
- Help

- Prev Class
- Next Class

- Frames
- No Frames

- All Classes

- Summary:
- Nested |
- Field |
- Constr |
- Method

- Detail:
- Field |
- Constr |
- Method


org.workflowsim.clustering.balancing

## Class BalancedClustering

- java.lang.Object
- - org.workflowsim.clustering.BasicClustering
  - - org.workflowsim.clustering.balancing.BalancedClustering

- All Implemented Interfaces:
  :   ClusteringInterface

  ---

    

  ```
  public class BalancedClustering
  extends BasicClustering
  ```

  BalancedClustering is a clustering method that aims balancing task runtime
  and data dependency. All BalancedClustering methods should extend it

  Since:
  :   WorkflowSim Toolkit 1.0

- - ### Constructor Summary

    Constructors

    Constructor and Description

    `BalancedClustering(int clusterNum)`

    Initialize a BalancedClustering method
  - ### Method Summary

    Methods

    | Modifier and Type | Method and Description |
    | --- | --- |
    | `void` | `cleanTaskSetChecked()` Clean the checked flag of a taskset |
    | `java.util.Map<java.lang.Integer,java.util.ArrayList<TaskSet>>` | `getCurrentTaskSetAtLevels()` Gets the current tasks per level |
    | `void` | `printMetrics()` Print out all the balancing metrics |
    | `void` | `run()` The main function of BasicClustering |

    - ### Methods inherited from class org.workflowsim.clustering.BasicClustering

      `addClustDelay, addRoot, addTasks2Job, addTasks2Job, clean, getJobList, getTask2Job, getTaskFiles, getTaskList, setTaskList, updateDependencies`
    - ### Methods inherited from class java.lang.Object

      `clone, equals, finalize, getClass, hashCode, notify, notifyAll, toString, wait, wait, wait`

- - ### Constructor Detail


    - #### BalancedClustering

      ```
      public BalancedClustering(int clusterNum)
      ```

      Initialize a BalancedClustering method

      Parameters:
      :   `clusterNum` - clusters.num
  - ### Method Detail


    - #### cleanTaskSetChecked

      ```
      public void cleanTaskSetChecked()
      ```

      Clean the checked flag of a taskset


    - #### printMetrics

      ```
      public void printMetrics()
      ```

      Print out all the balancing metrics


    - #### getCurrentTaskSetAtLevels

      ```
      public java.util.Map<java.lang.Integer,java.util.ArrayList<TaskSet>> getCurrentTaskSetAtLevels()
      ```

      Gets the current tasks per level

      Returns:
      :   tasks list per level


    - #### run

      ```
      public void run()
      ```

      **Description copied from class: `BasicClustering`**

      The main function of BasicClustering

      **Specified by:**
      :   `run` in interface `ClusteringInterface`

      **Overrides:**
      :   `run` in class `BasicClustering`


- Overview
- Package
- Class
- Use
- Tree
- Deprecated
- Index
- Help

- Prev Class
- Next Class

- Frames
- No Frames

- All Classes

- Summary:
- Nested |
- Field |
- Constr |
- Method

- Detail:
- Field |
- Constr |
- Method
